# Rearing system and immune status influence the small intestinal microbiota of IPB-D3 chickens: A full-length 16S rRNA metagenomic approach

**DOI:** 10.14202/vetworld.2025.2206-2221

**Published:** 2025-08-02

**Authors:** Wawan Kuswandi, Cahyo Budiman, Isyana Khaerunnisa, Cece Sumantri

**Affiliations:** 1 Department of Animal Production and Technology, Faculty of Animal Science, IPB University, Bogor, Indonesia; 2 Biotechnology Research Institute, Universiti Malaysia Sabah, Kota Kinabalu, Sabah, Malaysia; 3 Research Center for Applied Zoology, National Research and Innovation Agency (BRIN), Bogor, Indonesia

**Keywords:** 16S ribosomal RNA, free-range, gut microbiome, IPB-D3 chicken, leukocyte, rearing system, intestinal health

## Abstract

**Background and Aim:**

The small intestinal microbiota plays a pivotal role in poultry digestion and immune function. Rearing systems can influence their composition, thereby affecting the overall health and performance of the birds. This study aimed to investigate how rearing systems (intensive [IN] vs. free-range [FR]) and immune status, reflected by leukocyte profiles, influence the small intestinal microbiome of IPB-D3 chickens, a genetically improved Indonesian local breed.

**Materials and Methods:**

Ninety IPB-D3 chickens were reared for 12 weeks under either IN or FR systems. Hematological profiling was conducted to assess health status, with leukocyte counts used to stratify birds. Microbiota samples from the small intestine were analyzed using full-length 16S ribosomal RNA (V1–V9) sequencing on the Oxford Nanopore platform. Taxonomic identification was performed using the SILVA database. Statistical comparisons were made using t-tests, and microbial diversity was assessed through alpha and beta diversity metrics.

**Results:**

While most hematological parameters did not differ significantly between rearing systems, total leukocyte counts were higher in intensively reared chickens (p = 0.002). FR chickens exhibited significantly greater microbial diversity (p < 0.05) across multiple alpha diversity indices. A total of 1,294 unique species were identified in FR birds versus 720 in the IN group, with 1,761 shared species. Leukocyte level further influenced microbial profiles; chickens with high leukocyte (HL) counts were dominated by *Ligilactobacillus aviarius*, whereas low-leukocyte chickens had a higher abundance of *Bacteroides caecigallinarum. Gallibacterium anatis*, a potential pathogen, dominated in IN systems with elevated leukocytes.

**Conclusion:**

This study demonstrates that both the rearing environment and immune status substantially influence small intestinal microbial composition in IPB-D3 chickens. FR systems promoted richer, more beneficial microbial communities, while IN systems, especially with HL levels, were associated with opportunistic pathogens. Leukocyte profiling may serve as a non-invasive biomarker for gut health, supporting future development of precision poultry management strategies and immune-responsive probiotics.

## INTRODUCTION

The gastrointestinal tract harbors a diverse array of bacterial species that play a crucial role in maintaining the health and productivity of livestock. These microbes facilitate the digestion of complex nutrients and the synthesis of bioactive compounds, such as short-chain fatty acids (SCFAs), which improve energy efficiency and support immune function [1]. SCFAs produced by commensal gut microbes are particularly important in modulating immune responses, including the regulation of leukocyte activity and the control of inflammation. The gut microbiota establishes a competitive barrier against pathogens [2], promotes immune system maturation [3], enhances intestinal barrier integrity [4], and modulates both innate and adaptive immune responses [5]. In chickens, microbial communities are dispersed throughout the gastrointestinal tract, with the small intestine exhibiting one of the highest concentrations and microbial complexities [6, 7]. The small intestine serves not only as the primary site for digestion and nutrient absorption but also plays a vital role in mucosal immune responses. The small intestine has become a central focus in poultry microbiome research because of its dual function.

The microbial diversity in this region is influenced by environmental conditions, rearing systems, dietary practices, antibiotic administration, and stress, all of which have a direct impact on poultry health and productivity. Understanding microbial dynamics in the small intestine is essential for evaluating gut functionality and immunological competence, particularly in high-performance poultry breeds. The diversity of gut microbiota in chickens is profoundly influenced by the rearing system, with free-range (FR) and intensive (IN) environments exerting distinct impacts on microbial composition [8]. In the FR system, chickens have access to a more natural outdoor environment, which enriches their gut microbiome through exposure to soil, plants, and insects. Consequently, FR chickens generally exhibit greater diversity in the microbiome [9, 10]. Conversely, in IN rearing systems, chickens are kept in confined spaces with a more controlled environment. Such limited environmental exposure may reduce microbial diversity owing to restricted interaction with exogenous microbial populations [11]. In IN rearing systems, suboptimal conditions, including overcrowding, substandard hygiene, insufficient ventilation, and limited mobility, can induce stress; elevate pathogen susceptibility; and disrupt gut microbial equilibrium [12].

The diversity of gut microbiota, which encompasses both pathogenic and commensal organisms, is critical to the immunophysiological responses of chickens [13]. While rearing systems significantly influence microbial composition, the health status of chickens is equally important in shaping the gut microbiome. When non-pathogenic microbes are dominant, they function as probiotics, promoting gut health and strengthening immune resilience. Conversely, a higher presence of pathogenic microbes can trigger excessive immune responses, leading to chronic inflammation and physiological disorders [14]. Leukocytes, particularly neutrophils, play a crucial role in host defense through phagocytosis, and elevated leukocyte counts often indicate immune activation in response to a pathogenic challenge [15]. Therefore, hematological parameters are reliable indicators of poultry health status. Advances in microbiome research, particularly through 16S ribosomal RNA (rRNA) gene sequencing, allow for in-depth analysis of gut microbial communities, including their diversity, function, and the effects of environmental and health-related factors [16]. These insights support the development of improved management strategies, reduce antibiotic dependency, and contribute to sustainable enhancements in poultry health and productivity [17]. Nonetheless, a paucity of studies examining gut microbiome profiles across diverse poultry species under varied rearing systems and health statuses remains, hindering the formulation of robust conclusions and the development of generalized knowledge. This is especially true for indigenous poultry breeds in various countries, including Indonesia, where unique genetic backgrounds, distinct health characteristics, and diverse management practices may contribute to distinctive gut microbiome profiles. Accordingly, further investigation is warranted to elucidate these variations and to facilitate the development of sustainable, context-specific poultry production strategies.

The IPB-D3 chicken represents a newly developed local breed in Indonesia, selectively bred to improve the productivity and environmental adaptability of indigenous poultry. It is a selectively bred strain derived from the seventh-generation IPB D1 chicken and was officially released as an Indonesian local breed in 2019 [18], through Decree No. 693/KPTS/PK.230/M/9/2019. This breed was developed by crossing male Pelung-Sentul with female Kampung-Broiler chickens, each contributing 25% of the genetic composition. The IPB-D3 chicken inherited superior traits from both parental lines, including rapid growth and good meat quality [19], with males reaching 1,256 g and females reaching 1,042 g at 10 weeks of age. As a genetically improved local breed, the IPB-D3 chicken offers significant potential for enhancing poultry production in Indonesia, especially in smallholder and semi-IN systems.

In chickens, digestive efficiency and a balanced gut microbiome significantly influence growth performance, both of which are essential for nutrient assimilation and immune competence [20]. The IPB-D3 chicken exhibits good environmental adaptability [21] and strong disease resistance, including high immunoglobulin Y (IgY) levels of up to 10.10 μg/mL [22], primarily due to the crossbreeding of local and commercial lines. The gut microbiome of IPB-D3 chickens is likely to exhibit distinct characteristics given its superior genetics and adaptability. However, limited data exist on the gut microbial composition, particularly under varying rearing conditions. Considering the microbiota’s crucial role in digestion, immunity, and overall performance, investigating the gut microbiome profile of IPB-D3 chickens is essential. Studies on laying hens have demonstrated that rearing systems have a significant impact on gut microbiota diversity. It has been reported that hens raised in open (FR) systems exhibit greater microbial diversity than those kept in IN environments [23]. Similarly, beneficial bacteria predominantly colonized FR chickens at the genus level, including *Coprococcus, Clostridium, Butyricimonas, Paraprevotella*, and *Acinetobacter* [24]. The abundance and diversity of these bacteria, particularly in the small intestine, were significantly higher in FR chickens than in caged chickens.

In contrast, the microbial diversity of caged chickens was primarily dominated by *Bacillus*. However, no research has specifically explored the gut microbiome composition of high-performing local breeds, such as the IPB-D3 chicken, particularly in relation to different rearing systems. Given the pivotal role of the gut microbiota in digestion, immunity, and productivity, profiling the gut microbiome of IPB-D3 chickens is both relevant and necessary. Therefore, this study aimed to investigate the small intestinal microbiome of IPB-D3 chickens reared under different systems, with specific emphasis on health status as indicated by leukocyte profiles. For the first time, the 16S metagenomic approach was applied to investigate the gut microbiota composition of this chicken line, with a specific focus on the small intestine, a critical site for metabolic efficiency and mucosal immune responses. The 16S rRNA gene contains nine hypervariable regions (V1–V9) that encode taxonomic signatures essential for accurate bacterial identification [25]. In this study, full-length 16S rRNA gene sequencing was employed, enabling taxonomic classification down to the species level and comprehensive characterization of microbial diversity. Unlike conventional 16S rRNA methods, which typically resolve taxonomy only to the genus level, Oxford Nanopore Technology enables species-level resolution, providing deeper insights into host–microbiota interactions [26]. However, species-level data on the gut microbiota of improved indigenous chicken breeds in Indonesia, including the IPB-D3 line, are limited.

While numerous studies have documented the influence of rearing systems on gut microbiota diversity in commercial poultry, a critical lack of species-level microbial profiling remains in genetically improved local chicken breeds, particularly in Southeast Asia. Existing literature has primarily focused on broilers and laying hens, often using genus-level resolution through conventional 16S rRNA sequencing, which limits the detection of subtle yet impactful microbial variations. Moreover, the interactive effects of immune status, specifically leukocyte profile, on gut microbiota composition remain underexplored. In Indonesia, the IPB-D3 chicken has been developed as a high-performing, adaptable local breed, yet its intestinal microbiome under different rearing environments has not been investigated. Given that both environmental exposure and immunological responses can significantly influence gut microbial ecosystems, the absence of detailed microbiome data in this context hampers the development of precision health and productivity strategies tailored to local breeds.

This study aims to characterize the small intestinal microbiome of IPB-D3 chickens raised under IN and FR systems, with a particular focus on how leukocyte levels, serving as indicators of immune status, influence microbial diversity and composition. Utilizing full-length 16S rRNA gene sequencing with Oxford Nanopore Technology, this study seeks to achieve species-level resolution of gut microbial communities. The findings are expected to elucidate the interplay between rearing conditions, host immune response, and gut microbiota, thereby informing the development of immune-based monitoring tools, microbial interventions, and sustainable rearing practices for genetically improved local poultry breeds.

## MATERIALS AND METHODS

### Ethical approval

All animal management procedures and experimental protocols were conducted in accordance with national and international animal welfare guidelines, including the ARRIVE guidelines and those set by the World Organization for Animal Health (WOAH), and were approved by the Animal Ethics Committee of the School of Veterinary Medicine and Biomedical Sciences, IPB University (Approval No. 209/KEH/SKE/IV/2024).

### Study period and location

The study was conducted between January and November 2024. IPB-D3 chickens were reared at the Field Laboratory of Animal Breeding and Genetics, Faculty of Animal Science, IPB University, Dramaga, Bogor, located at approximately 250 m above sea level. Hematological analyses were performed at the IPB University Laboratory of Meat and Working Animal Nutrition. DNA extraction was conducted at the Genomics Laboratory, National Research and Innovation Agency (BRIN), Cibinong, which operates under Biosafety Level 1 standards. Subsequently, the amplified polymerase chain reaction (PCR) products were sequenced using Oxford Nanopore Technologies (ONT) at PT Genetika Science Indonesia.

### Animal management and experimental design of the animals

**Figure 1:**
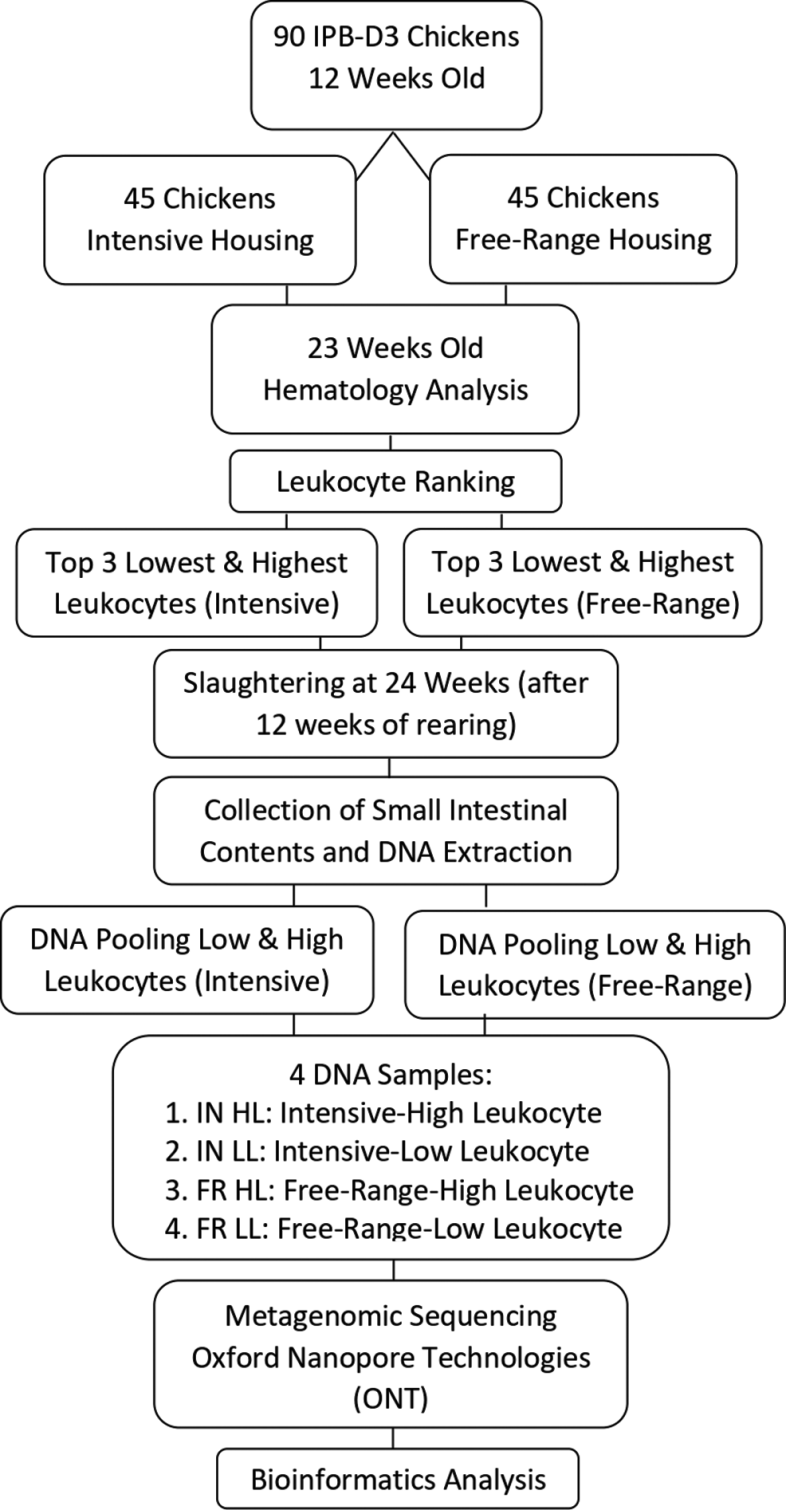
Workflow for intestinal microbiome profiling in IPB-D3 chickens based on rearing systems and leukocyte classification from hematological ranking.

Ninety 12-week-old IPB-D3 chickens were used to assess the small intestine microbiome under two rearing systems. Birds were randomly assigned into two groups using simple randomization: IN and FR systems, with 45 chickens per group. Each group consisted of 21 males and 24 females, resulting in an approximately 1:1 male-to-female ratio. Chickens were reared for 12 weeks (Figure 1). The initial body weight of chickens in the IN system was 619.82 ± 114.42 g, whereas that of chickens in the FR system had an initial body weight of 625.11 ± 109.02 g. Chickens were fed a commercial diet twice daily. The housing was equipped with feeders, drinkers, perching areas, and rice husk bedding. Both the IN and FR housing systems were cleaned according to standard procedures, without the use of disinfectants, before placement. The ambient temperature and humidity were monitored at 6:00 a.m., 12:00 p.m., and 5:00 p.m. daily using adigital thermohygrometer (HTC-1, Equipslab, China). These management procedures ensured a standardized baseline across both systems while allowing environmental differences inherent to the rearing design to influence the physiological and microbial responses of the birds. This setup was intended to reflect realistic production settings while maintaining consistency in nutrition, hygiene, and population structure across the experimental groups.

### Preparation of the FR rearing system

The FR rearing area measured 20 m × 10 m. It featured natural vegetation, including banana trees, Indigofera, and wild grasses, and was enclosed with netting to prevent chickens from escaping and to protect them from predators. The system followed Australian FR standards, maintaining a maximum stocking density of one chicken per square meter [27]. The chickens were free to move within the area, allowing them to exhibit natural behaviors, such as foraging, scratching the ground, and dust bathing, which promote their well-being and health. The area was also equipped with poles or small trees to support roosting behavior. In the FR system, chickens were released between 6:00 a.m. and 5:00 p.m. and had access to shaded areas and naturally growing vegetation, such as Indigofera and wild grasses. No additional vegetation was planted, except for resting areas (perches). At 5:00 p.m., the chickens were returned to an indoor enclosure (4 m × 3 m) to protect them from nocturnal predators. Forty-five IPB-D3 chickens were housed under this FR system (Figure 1).

### Preparation of the IN rearing system

The IN rearing system used a 6 m × 1.5 m enclosure designed to provide the chickens with optimal conditions. It used a conventional open-sided house without automated climate control equipment, such as fans, heaters, or humidifiers. Therefore, the environmental conditions (temperature and humidity) were allowed to fluctuate naturally and were not actively regulated. The housing featured adequate ventilation to support airflow and a floor design that facilitated easy cleaning, which helped prevent waste accumulation and reduce disease risk. The stocking density was managed to minimize stress and ensure animal comfort. Forty-five IPB-D3 chickens were housed in this IN system (Figure 1).

### Sample collection

#### Blood sample collection

At 23 weeks of age, 2 mL of blood was collected from the brachial vein using a 3 mL syringe fitted with a 23-gauge needle (0.6 mm × 30 mm). Blood sampling was performed without anesthesia; only 70% alcohol was applied to the skin surface for disinfection before venipuncture. The collected blood was transferred into ethylenediaminetetraacetic acid-coated tubes and gently inverted in a figure-eight motion to ensure proper mixing with the anticoagulant. Hematological analysis was conducted according to the protocol described by Onunkwo *et al*. [28], which included measurements of packed cell volume (PCV), erythrocyte sedimentation rate (ESR), hemoglobin concentration (Hb), and total counts of white blood cells (WBC) and red blood cells (RBC). Differential leukocyte counts were also performed, covering lymphocytes (LYM), heterophils (HET), eosinophils (EOS), monocytes (MON), and basophils (BAS), using manual counting with a hemocytometer and standard staining techniques. Erythrocyte indices were calculated, including mean corpuscular volume (MCV), mean corpuscular hemoglobin (MCH), and mean corpuscular hemoglobin concentration (MCHC), using the formulas: MCV = (PCV × 10)/RBC, MCH = (Hb × 10)/RBC, and MCHC = (Hb × 100)/PCV. The heterophil-to-lymphocyte (H/L) ratio was determined as a physiological stress indicator using the formula H/L = number of HET/number of LYM. These hematological parameters offer valuable insights into the physiological condition, stress level, and immunological status of chickens reared in various systems.

#### Leukocyte-based selection of chickens for gut microbiota sampling

Chickens were selected based on hematological findings, with a particular focus on total leukocyte levels. Following the completion of hematological analysis, particularly the total leukocyte count, individual chickens were ranked from the highest to the lowest leukocyte levels within each treatment group. Six unsexed chickens were selected from each rearing group without regard to sex distribution, as the selection was based exclusively on leukocyte count. Each subset consisted of three birds with the highest and three with the lowest leukocyte values, determined solely by numerical ranking rather than a predefined threshold or reference cutoff (Table 1). This method was chosen to reflect the natural variability in individuals’ immune status. The sample size was determined based on methodological considerations to represent two distinct physiological immune states within each rearing system. Additionally, this number was deemed sufficient for the initial exploratory profiling of the gut microbiota using Oxford Nanopore sequencing, ensuring adequate sequencing depth and species-level resolution. The selected chickens were euthanized in accordance with animal welfare guidelines, and the small intestine was isolated by aseptic dissection. Microbiota samples were collected from the entire small intestinal tract, including the duodenum, jejunum, and ileum. The intestinal contents were gently scraped using a sterile spatula, pooled across all segments, homogenized, and transferred into tubes containing DNA/RNA Shield to preserve microbiome integrity.

**Table 1:** Summary of raw data generated from 16s ribosomal RNA metagenomic sequencing of bacterial communities in the small intestine of IPB-D3 chickens under different rearing systems.

Rearing system	Leukocyte-based grouping	Group	n	Average total leukocyte count (10^3^ mm^-3^)	Raw data		Filtered data	
	
					Number of reads (bases)	Mean read length (bp)	Number of reads (bases)	Mean read length (bp)
Intensive	High	IN-HL	3	35.38	115.081	1449.5	85.903	1610.6
	Low	IN-LL	3	7.15	116.161	1467.0	95.702	1605.9
Free-range	High	FR-HL	3	28.29	123.057	1364.0	89.991	1619.6
	Low	FR-LL	3	6.79	118.104	1432.7	93.823	1601.3

IN=Intensive, FR=Free-range, HL=High leukocyte level, LL=Low leukocyte level, n=Number of samples. Chickens were categorized based on total leukocyte counts before microbiota analysis

### DNA extraction

DNA was extracted from small intestinal content samples (n = 12) using the ZymoBIOMICS DNA Mini Kit (Zymo Research, CA, USA, Cat. No. D4300, Lot No. 224411), which was used to extract DNA from small intestinal content samples (n = 12) according to the manufacturer’s instructions. Each sample (750 μL), preserved in DNA/RNA Shield (Zymo Research), was homogenized with 750 μL of lysis buffer in a ZR BashingBead Lysis Tube using a TissueLyser III (Qiagen, China) at 30 Hz for 1 min across five cycles with 5-min intervals. After centrifugation at 10,000 × *g* for 1 min, the supernatant was filtered using a Zymo-Spin III-F Filter. The filtrate was mixed with a DNA Binding Buffer and applied to a Zymo-Spin IICR Column for binding and sequential washing using DNA Wash Buffers 1 and 2. DNA was eluted in 100 μL of DNase/RNase-free water and further purified using a Zymo-Spin III-HRC Filter with HRC Prep Solution. DNA concentration and purity were assessed using a NanoDrop 2000 and Qubit Fluorometer (Thermo Scientific), while DNA integrity was evaluated using 1% agarose gel electrophoresis. The extracted DNA was stored at −80°C until further analysis. No negative or positive controls were included during the DNA extraction process; however, to minimize potential contamination, all procedures were performed using sterile techniques.

### Amplicon library preparation and sequencing analysis

Equal concentrations of 12 DNA samples were pooled into four groups representing IN and FR systems with high and low leukocyte levels, respectively. Microbiome profiling was conducted using full-length 16S rRNA (V1–V9) amplicon sequencing with primers 27F (5′-AGAGTTTGATCMTGGCTCAG-3′) and 1492R (5′-GGTTACCTTGTTACGACTT-3′), as described by Kim *et al*. [29]. PCR amplification was performed using KOD Multi & Epi (Toyobo, Japan) in a 25 μL reaction containing 0.3 μL DNA, 0.15 μL each of forward and reverse primers, and 12.5 μL of KOD enzyme mix. PCR cycling conditions were 95°C for 1 min, followed by 35 cycles at 95°C for 15 s (denaturation), 60°C for 10 s (annealing), and 72°C for 10 s (extension). PCR products were analyzed by 1% agarose gel electrophoresis and visualized using an ultraviolet transilluminator. Successfully amplified polymerase chain reaction products were purified using AMPure XP magnetic beads (Beckman Coulter). The DNA library was quantified using the Qubit dsDNA High Sensitivity Assay Kit (Thermo Fisher Scientific) according to the manufacturer’s protocol. Sequencing was performed using the Oxford Nanopore PromethION platform with an R10.4.1 flow cell and the Native Barcoding Kit 24 V14 (SQK-NBD114.24, ONT). Following quality filtering to exclude low-quality sequences, the average number of high-quality, analyzable reads per sample was calculated and is presented in Table 1. Libraries passing quality control were sequenced using full-length 16S rRNA kits (ONT) on a Nanopore sequencer, and microbial profiles were analyzed through downstream bioinformatic pipelines.

### Bioinformatics analysis

Following sequencing, bioinformatic analysis was performed using ONT with the PromethION device, operated through the MinKNOW software for real-time data acquisition in FAST5 format. The data were then converted into FASTQ format through base calling using Dorado with a high-accuracy model to enhance the reliability of the base calling. Data quality was assessed using NanoPlot to visualize read length and quality distribution, while NanoFilt was used to filter reads based on a minimum read length of 1000 bp and a minimum average quality score of Q7. Filtered reads were analyzed using a centrifuge for taxonomic classification against the SILVA 16S rRNA reference database (release 138). A minimum Centrifuge confidence score threshold of 0.7 was applied to ensure reliable taxonomic assignments. The taxonomic profiles were further explored using Avian for interactive visualization and KronaTools to generate radial taxonomy charts. Alpha and beta diversity metrics, including the Shannon index and Bray–Curtis dissimilarity, were calculated using the phyloseq and vegan packages in R (RStudio). Additional analyses, such as principal coordinate analysis (PCoA), heatmaps, and taxonomic barplot, were also conducted in R based on microbial composition. No statistical tests for differential abundance or group comparisons were performed; thus, no corrections for multiple comparisons such as false discovery rate (FDR) were applied in this study.

### Statistical analysis

A bioinformatic approach was employed for microbiome analysis, which began with base calling, sequence quality filtering, and taxonomic identification using the SILVA 16S rRNA reference database. The processed data were then analyzed to calculate alpha diversity indices (Observed, Chao1, ACE, Shannon, Simpson, Inverse Simpson, and Fisher) and beta diversity using Bray–Curtis dissimilarity, which was visualized through PCoA. All analyses and visualizations were performed using RStudio (R version 4.2.3). In addition, blood profile data, temperature, and environmental humidity were analyzed using independent t-tests after confirming statistical assumptions using the Statistical Package for the Social Sciences version 26 (IBM Corp., NY, USA).

## RESULTS

### Environmental conditions and climate monitoring during rearing

The average midday temperature in the FR system (34.56°C) was significantly higher than that in the IN system (33.46°C; P = 0.006), while the midday humidity was significantly lower in the FR system (62.93%) than in the IN system (67.69%; P = 0.006). The schematic layout of the experimental design, housing conditions, and chicken distribution is presented in Table 2. These findings indicate that the FR system tends to be warmer and drier during the day, which may influence animal comfort and microbial exposure.

**Table 2:** The environmental temperature and humidity conditions were observed throughout the rearing period under intensive and free-range systems.

Time of Day (UTC + 7)	Intensive (x̄ ± sd)	*Free-range* (x̄ ± sd)	p-value
Temperature (°C)			
Morning (06.00 AM)	25.23 ± 1.10	25.10 ± 1.68	0.521^ns^
Noon (12.00 PM)	33.46 ± 2.63	34.56 ± 3.06	0.006*
Afternoon (05.00 PM)	28.39 ± 2.20	27.98 ± 2.08	0.169^ns^
Humidity (%)			
Morning (06.00 AM)	90.32 ± 5.04	90.43 ± 6.02	0.891^ns^
Noon (12.00 PM)	67.69 ± 11.89	62.93 ± 13.12	0.006*
Afternoon (17.00 PM)	81.70 ± 6.65	81.17 ± 6.86	0.575^ns^

x̄=Mean, sd=Standard deviation, p-value=t-test significance between chickens raised in intensive and free-range systems,

* Significantly different (p < 0.05); ns=not significantly different

### Hematological parameters of IPB-D3 chickens under different rearing systems

As shown in Table 3 [30, 31], hematological parameters, including total erythrocyte count, hematocrit, hemoglobin concentration, and erythrocyte indices (MCV, MCH, and MCHC), did not differ significantly (p > 0.05) between chickens raised in IN and FR systems. All values remained within normal physiological ranges, suggesting that both rearing systems adequately maintained hematological homeostasis in IPB-D3 chickens. The total erythrocyte count was marginally lower in intensively reared chickens (2.78 ± 0.64 × 10^6^/mm^3^) compared to their FR counterparts (2.96 ± 0.63 × 10^6^/mm^3^), but this difference was not statistically significant (p = 0.269). Hemoglobin levels were higher in FR chickens (9.71 ± 1.44 g/dL) than in intensively reared chickens (8.99 ± 1.26 g/dL), although the difference was not statistically significant (p = 0.060). The consistency in erythrocyte and hemoglobin levels implies that both rearing systems provided adequate environmental and nutritional support for oxygen transport and metabolic efficiency. Thus, differences in rearing systems did not adversely affect the physiological stability of IPB-D3 chickens, particularly in terms of erythrocyte integrity and oxygen-carrying capacity.

**Table 3:** Erythrocyte, hematocrit, hemoglobin, and erythrocyte indices in IPB-D3 chicken blood under different management systems.

Parameters	IPB-D3 Chicken		p-value	Normal

	Intensive (x̄ ± sd)	Free-range (x̄ ± sd)		
Erythrocyte (10^6^/mm^-3^)	2.78 ± 0.64	2.96 ± 0.63	0.269^ns^	2.5–3.9[30]
Hematocrit (%)	28.48 ± 3.84	29.51 ± 3.87	0.285^ns^	24–43[30]
Hemoglobin (g/dL)	8.99 ± 01.26	9.71 ± 1.44	0.060^ns^	10.2–15.1[30]
Erythrocyte indices				
MCV (fL)	106.05 ± 22.12	103.31 ± 21.56	0.614^ns^	90–140 [31]
MCH (pg)	33.62 ± 6.53	33.88 ± 6.86	0.878^ns^	33–47 [31]
MCHC (%)	32.00 ± 3.89	32.97 ± 2.98	0.249^ns^	26–35 [30]

x̄=Mean; sd=Standard deviation, p-value=t-test significance between chickens raised in intensive and free-range systems; ns=Not significantly different. MCV=Mean corpuscular volume, MCH=Mean corpuscular hemoglobin, MCHC=Mean corpuscular hemoglobin concentration

As indicated in Table 4 [30, 32], the total leukocyte count differed significantly (p = 0.002), with intensively reared chickens exhibiting higher counts (20.12 ± 9.03 × 10^3^/mm^3^) compared with FR chickens (14.27 ± 6.28 × 10^3^/mm^3^). Despite the observed difference, leukocyte values remained within the normal reference range (6–40 × 10^3^/mm^3^), indicating adequate immune support under both rearing systems. No significant differences were noted in differential leukocyte parameters, including LYM, HET, EOS, MON, and BAS (p > 0.05). Likewise, the H/L ratio was comparable between groups, suggesting no significant stress induced by the differing rearing environments. Overall, the rearing system had a significant impact on only the total leukocyte count, with higher values observed in intensively reared chickens. However, no substantial differences in leukocyte subtypes or H/L ratios were observed, indicating the absence of marked immunological disturbances across rearing systems.

**Table 4:** Leukocyte parameters, leukocyte differential, and H/L ratio in chickens using intensive and free-range management systems.

Parameters	IPB-D3 Chicken		p-value	Normal

	Intensive (*x̄* ± sd)	Free-range (x̄ ± sd)		
Leukocyte (10^3^ mm^-3^)	20.12 ± 9.03	14.27 ± 6.28	0.002*	06–40 [32]
Leukocyte deferential				
Lymphocyte (%)	49.02 ± 7.77	48.88 ± 8.04	0.940^ns^	20–84 [32]
Heterophil (%)	37.25 ± 7.45	37.97 ± 8.36	0.718^ns^	9–56 [32]
Eosinophils (%)	8.46 ± 2.16	7.63 ± 2.51	0.163^ns^	0–7[30]
Monocytes (%)	4.42 ± 2.15	4.70 ± 1.58	0.544^ns^	2–8 [32]
Basophils (%)	0.84 ± 0.07	0.82 ± 0.06	0.396^ns^	0–1[30]
H/L (%)	0.80 ± 0.31	0.83 ± 0.32	0.775^ns^	0.2–0.8[30]

x̄: mean; sd: Standard Deviation; p-value=Significance of the t-test between chickens raised in intensive and free-range systems;

* Significantly different (p < 0.05); ns: Not significantly different. H/L=Heterophil/lymphocyte ratio

### Overview of raw sequencing data quality

Raw data were generated by sequencing the V1–V9 region of the 16S rRNA gene, capturing both total read counts and average base lengths before filtration. As presented in Table 1, the raw sequencing data revealed an average sequence length of 1,428.3 bp, which was slightly shorter than the target length of 1,500 bp. However, this minor deviation did not compromise the overall sequence quality. After the filtration process, which eliminates low-quality sequences, the number of sequence reads decreased, as expected. The average sequence length increased to 1,605.93 bp after filtration, slightly exceeding the target but remaining within an acceptable range for downstream analysis. This indicates that the filtration process effectively enriched the data by retaining higher-quality sequences and improving the reliability of the microbiome profile. The table provides a breakdown of the raw and filtered data for each leukocyte group across both rearing systems. The values demonstrate the influence of the filtration process on both the quantity and quality of the sequence reads, highlighting that the overall sequence length improved while the number of reads decreased after filtration. This indicates that the filtration process enriched the dataset by enhancing the read quality, thereby supporting the more accurate characterization of the small intestinal microbiota in IPB-D3 chickens.

### Alpha diversity of gut microbiota in IPB-D3 chickens

**Figure 2:**
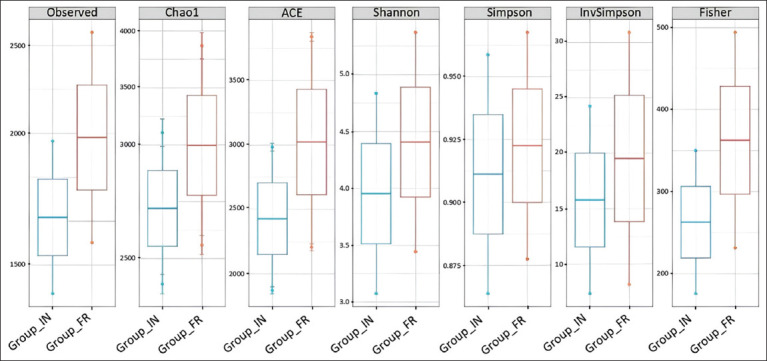
Alpha diversity revealed variations in the gut microbial community of chickens under two rearing systems, intensive (IN) and free-range (FR), as measured by (a) observed OTUs, Chao1, ACE, and Shannon indices and (b) Simpson, Inverse Simpson, and Fisher indices.

The alpha diversity indices for both the IN and FR systems exhibited distinct distribution patterns (Figure 2). The FR group demonstrates higher species richness than the IN group, as indicated by the observed operational taxonomic unit (OTU), Chao1, abundance-based coverage estimator (ACE), Shannon, Simpson, Inverse Simpson, and Fisher, which reflect a greater number of species in this system. The analysis consistently demonstrated a significantly higher diversity (p < 0.05) in the FR group compared to the IN group across all indices examined. The Observed Species (OTU) index, which represents the number of directly observed species, indicated that chickens raised under the FR system had significantly greater microbial diversity (p < 0.05), with a median and range of approximately 1300–2600, compared to 1000–2000 in the IN group. This suggests a broader environmental microbial exposure in the FR system. The Chao1 index, which estimates true species richness by accounting for rare species, also showed a significant difference (p < 0.05), with the FR group displaying a median richness of approximately 3000 and a spread approaching 4000, whereas the IN group had a median around 2600 and a lower spread under 3500. Similarly, the ACE index, which also focuses on species richness estimation, demonstrated a significantly higher value (p < 0.05) in the FR group, with a median of approximately 3100 (range: 2200–3800), compared to the IN group’s median of approximately 2500 (range: 1900–3000). Regarding the Shannon index, which incorporates both species richness and evenness, the FR group showed significantly higher diversity (p < 0.05) with a median value of approximately 4.4, compared to 3.9 in the IN group. The FR group also exhibited a broader diversity range (±3.5–5.3) compared to the IN group (±3.0–4.8), indicating a more balanced and complex microbial community. The Simpson index, which measures species dominance within the community, showed a significantly higher median value (p < 0.05) in the FR group (approximately 0.925) than in the IN group (approximately 0.910). The FR group also had a wider range (0.875–0.965) compared to IN (0.865–0.955), reflecting a more evenly distributed microbial community. The Inverse Simpson index, which gives more weight to overall community diversity, also revealed a significantly higher diversity (p < 0.05) in the FR group, with a median of approximately 20 and a range of 7–32, compared to the IN group’s median of approximately 16 and a range of 4–25. Finally, the Fisher index, which estimates log-series distribution parameters in complex microbial communities, showed a significantly higher value (p < 0.05) in the FR group (median approximately 370) compared to the IN group (median approximately 280). Collectively, these alpha diversity analyses confirm that compared to the IN system, the FR management system significantly supports the development of a richer, more balanced, and more stable gut microbial community in IPB-D3 chickens.

### Beta diversity analysis of gut microbiota in IPB-D3 chickens

**Figure 3:**
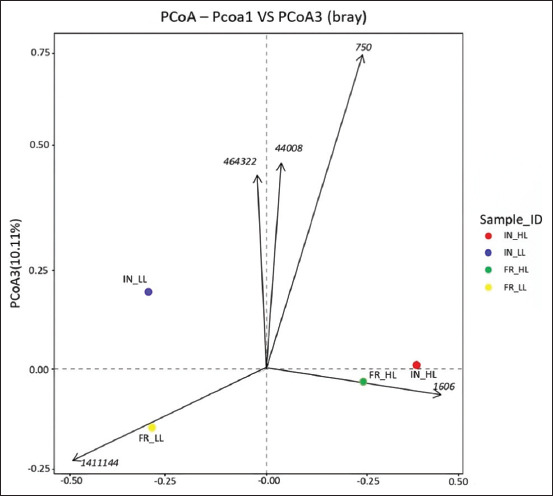
The principal coordinates analysis plot visualizes the distribution of small intestine samples across four groups IN-HL, IN-LL, FR-HL, and FR-LL to illustrate the differences in microbial diversity (beta diversity) among these groups. IN-HL=Intensive, high leukocyte count, IN-LL: Intensive, low leukocyte count, FR-HL=Free-range, high leukocyte count, FRLL=Free-range, low leukocyte count.

PCoA based on Bray–Curtis distances revealed that both housing system and leukocyte levels contributed significantly to the variation in gut microbiota communities of IPB-D3 chickens (p < 0.05). Figure 3 shows that the combination of PCoA axis 1 and axis 3, accounting for 70.16% and 10.11% of the total variation, respectively, provides detailed insights into community differences, allowing the identification of microbial shifts associated with immune responses or specific physiological conditions that may not be evident on other axis combinations. The four groups (IN_high leukocyte [HL], IN_low leukocyte [LL], FR_HL, and FR_LL) were color-coded to facilitate the visualization of distribution patterns and intergroup differences. Based on the distribution patterns on the plot, the gut microbial community structure of IPB-D3 chickens varied significantly (p < 0.05), influenced by both the rearing system (IN vs. FR) and leukocyte levels (high vs. low). PCoA1 captured the main variation significantly associated with housing system differences (p < 0.05), while PCoA3 reflected additional significant variation related to physiological status or leukocyte levels (p < 0.05). The PCoA1 versus PCoA3 plot showed that the IN_HL (red) and FR_HL (green) groups were closely clustered, suggesting that IPB-D3 chickens with high leukocyte (HL) levels may share similar microbiota structures, regardless of housing system, with no statistically significant difference observed between them (p > 0.05). In contrast, IN_LL (blue) and FR_LL (yellow) formed clearly separated clusters, indicating significant differences in microbiota composition (p < 0.05), likely due to the interaction between the housing system and LL status.

### Taxonomic profiling of the gut microbiome in IPB-D3 chickens

**Figure 4:**
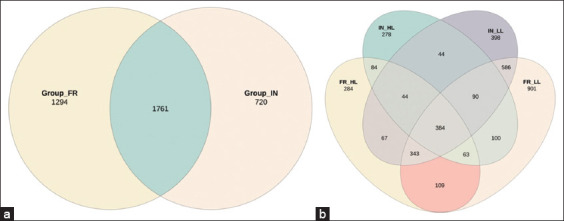
(a) Venn diagram showing the diversity of the small intestine microbiome in IPB-D3 chickens reared under free-range and intensive systems. (b) Venn diagram illustrating the distribution of the small intestine microbiome in IPB-D3 chickens based on the rearing system (intensive and free-range) and leukocyte levels (high and low).

Figure 4 shows that the relative diversity of the microbial community in the small intestinal digesta of IPB-D3 chickens is shown in Figure 4. Chickens raised under the FR system exhibit higher microbiome diversity than those in the IN system. A total of 1,294 unique microbial species were identified in the FR system compared with 720 in the IN system. Despite these disparities, 1,761 microbial species were shared across both systems, suggesting the presence of a stable core microbiota regardless of the rearing conditions (Figure 4a). Leukocyte levels also influence microbial diversity. Chickens with HL counts displayed reduced microbiome richness, with only 278 microbial species identified across both rearing systems. In contrast, the LL groups exhibited higher diversity, with 901 microbial species identified across both systems (Figure 4b). These findings suggest that leukocyte levels significantly impact microbiome diversity in addition to rearing conditions. Chickens with lower leukocyte counts tend to have a more diverse microbiome, which may have potential implications for their health and overall performance.

**Figure 5:**
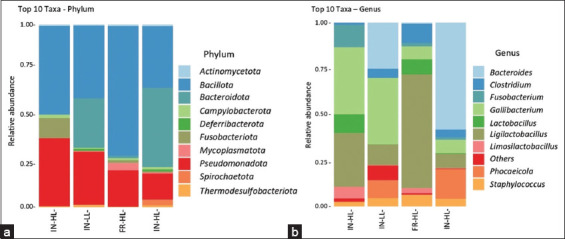
Relative abundance of gut bacteria in IPB-D3 chickens based on the rearing system and leukocyte levels. (a) Phylum-level composition. (b) Genus-level composition: IN-HL, INLL, FR-HL, FR-LL. IN-HL=Intensive, high leukocyte count, IN-LL: Intensive, low leukocyte count, FR-HL=Free-range, high leukocyte count, FR-LL=Free-range, low leukocyte count.

Based on metagenomic analysis (Figure 5a), the phylum *Bacillota* dominated across all groups, with the highest proportion observed in the FR_HL group (71%) and the lowest in FR_LL (33%). The IN_HL and IN_LL groups exhibited proportions of 49% and 38%, respectively. *Pseudomonadota* was also relatively dominant, particularly in the IN system, with IN_HL and IN_LL showing 37% and 28%, respectively, compared to lower levels in the FR system: 20% in FR_HL and 13% in FR_LL. *Bacteroidota* was highly abundant in FR_LL (40%) and IN_LL (26%) but was minimally abundant in FR_HL (1%) and IN_HL (0.2%). Overall, the variation in the dominance of *Pseudomonadota* and *Bacteroidota* suggests that the rearing system and leukocyte levels influence the composition of the gut microbial community. *Fusobacteriota* was notably present in IN_HL (11%) but remained below 1% in the other groups. *Spirochaetota* increased in FR_LL (3%), and *Mycoplasmatota* was most abundant in FR_HL (4%). Meanwhile, *Campylobacterota* appeared to be more dominant in the IN system, especially in IN_HL (2%), compared to other groups (0.5–1%). These findings indicate that the dominance of each phylum varies depending on the rearing system and leukocyte status.

Metagenomic analysis (Figure 5b) showed that the genus *Gallibacterium* was most dominant in chickens reared under IN systems, with relative abundances of 33% and 16% in IN_HL and IN_LL, respectively. In contrast, its abundance was lower in the FR groups, at 5% in FR_HL and 3% in FR_LL. The dominant genera in FR systems varied according to leukocyte levels, with *Ligilactobacillus* dominating in FR_HL (44%) and Bacteroides in FR_LL (20%). *Ligilactobacillus* was also prominent in IN_HL (26%) and tended to increase with higher leukocyte levels across all rearing systems. Bacteroides were more abundant in chickens with LL counts, particularly in the FR_LL (20%) and IN_LL (11%) groups.

**Figure 6:**
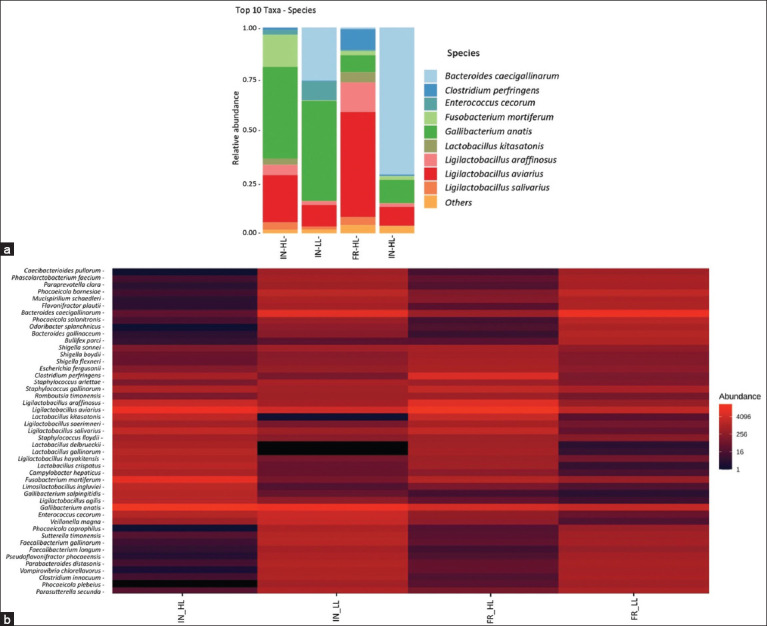
(a) Relative abundance of the 10 dominant bacterial species identified across different rearing systems based on leukocyte levels. (b) A heatmap illustrating the distribution patterns and abundance of bacterial species in each sample across different rearing systems: IN-HL, IN-LL, FR-HL, FR-LL. IN-HL=Intensive, high leukocyte count, IN-LL: Intensive, low leukocyte count, FR-HL=Free-range, high leukocyte count, FR-LL=Free-range, low leukocyte count.

Metagenomic profiling revealed the relative abundance of microbial species in the small-intestinal digesta of IPB-D3 chickens (Figure 6a). Dominant bacterial species differed across rearing systems, revealing distinct microbial distribution patterns within each experimental group. In intensively reared chickens with high leukocyte counts (IN_HL), the dominant bacterial species were *Gallibacterium anati*s (31%), *Ligilactobacillus aviariu*s (16%), *Fusobacterium mortiferum* (11%), *Lactobacillus araffinosu*s (3%), *Lactobacillus salivariu*s (3%), *Lactobacillus kitasatoni*s (2%), *Enterococcus cecorum* (2%), and *Clostridium perfringen*s (1%). In the IN_LL group, *G. anatis* remained dominant (16%), followed by *Bacteroides caecigallinarum* (9%), *L. aviarius* (3%), and *E. cecorum* (3%). In FR_HL chickens, *L. aviarius* was the dominant species (31%), followed by *L. araffinosus* (9%), *C. perfringens* (6%), *G. anatis* (5%), *L. kitasatonis* (3%), *L. salivarius* (2%), *F. mortiferum* (1%), *B. caecigallinarum* (1%), and *E. cecorum* (1%). In FR_LL chickens, *B. caecigallinarum* was the dominant species (16%), followed by *G. anatis* (3%) and *L. aviarius* (2%). These findings highlight the impact of rearing systems and leukocyte status on gut microbial composition, influencing community structure in distinct ways. At the species level, notable differences in small intestinal microbiota composition were observed based on the rearing system and leukocyte status. *G. anatis* (31%) was the dominant species in intensively reared chickens, while bacterial dominance varied according to leukocyte level in the FR group. *L. aviarius* (31%) predominated in FR chickens with HL counts, whereas *G. anatis* (3%) and *L. aviarius* (2%) exhibited comparable, lower abundances. Moreover, *L. aviarius* was more prevalent in chickens with elevated leukocyte counts across both rearing systems. Conversely, *B. caecigallinarum* was more abundant in chickens with LL counts than in those with elevated counts. These results indicate that leukocyte levels are associated with shifts in microbiota composition, with *L. aviarius* predominating in HL groups. *B. caecigallinarum* exhibited greater abundance in chickens with lower leukocyte counts.

### Heatmap visualization of dominant gut microbiota

The heatmap (Figure 6b) presents the relative abundance of the top 50 OTUs in the gut microbiota of IPB-D3 chickens across four treatment groups: IN_HL (IN system, HL count), IN_LL (IN system, LL count), FR_HL (FR, HL count), and FR_LL (FR, LL count). Red shading indicates high abundance, while black represents low abundance. Visualization highlights distinct microbial composition patterns shaped by both the rearing system and immune status. *G. anatis* was most abundant in the IN groups, especially in the IN_HL group. In contrast, *L. aviarius* dominated the FR_HL group, whereas *B. caecigallinarum* was enriched in the FR_LL group. Meanwhile, *Lactobacillus delbrueckii*, *Odoribacter splanchnicus*, and *Gallibacterium salpingitis* showed low abundance in specific groups.

## DISCUSSION

### Influence of rearing systems on gut microbial diversity and animal welfare

The rearing system is a critical determinant in poultry production, directly influencing health, productivity, gut microbiota diversity, and overall animal welfare. IPB-D3 chickens raised under FR conditions exhibited greater gut microbial diversity compared to intensively reared counterparts, likely due to enhanced environmental microbial exposure. Previous study by Cui *et al*. [23] has also shown that open environments promote the colonization of a wider range of microorganisms through broader ecosystem interactions. Increased microbial diversity is associated with improved immune resilience, enhanced nutrient metabolism, and a more stable intestinal ecosystem [33]. FR chickens often exhibit better physiological conditions, improved performance, and a lower risk of infection. Conversely, IN systems, which restrict microbial exposure, often result in more homogeneous microbiota that are prone to pathogen overgrowth and increased susceptibility to disease. Therefore, the rearing system plays a crucial role in maintaining the balance and stability of the gut microbiota, which in turn directly influences poultry health and productivity.

### Microbiota composition in the small intestine of chickens

The rearing system had a significant influence on the gut microbiota composition in chickens. FR chickens exhibited higher microbial heterogeneity than those raised intensively, attributable to increased interaction with diverse environmental microbes. This exposure contributes to a more complex and dynamic microbial community. In contrast, intensively reared chickens developed a more uniform microbiota, likely due to standardized housing conditions and homogeneous diets. These findings are consistent with those of a previous study by Varriale *et al*. [34], which suggested that interactions with the external environment in FR systems enhance gut microbiome diversity. This difference highlights how the rearing environment influences the composition of the microbiota and plays a crucial role in maintaining the stability of the microbial ecosystem in the digestive tract of poultry. These microbial differences are particularly evident in the small intestine, a region integral to both nutrient absorption and mucosal immune surveillance. Unlike the cecum, which harbors a dense and relatively stable microbial population for fermentation, the small intestine supports a fluctuating and responsive microbiota, making it highly sensitive to environmental changes, immune status, and dietary inputs. Thus, the small intestine is crucial for monitoring host-microbe–immune interactions, particularly in rearing systems.

### Physiological resilience and immunological adaptation in IPB-D3 chickens

IPB-D3 chickens have shown robust disease resistance, environmental adaptability, and high productivity across rearing systems. Their robust immune responses are reflected in elevated IgY levels, reaching up to 10.10 μg/mL [22]. Their ability to perform well under both IN and FR conditions reflects their adaptability, which is supported by a diverse gut microbiota that enhances digestion and pathogen resistance. Furthermore, IPB-D3 chickens maintain optimal production performance and efficient feed conversion, making them a promising local breed for sustainable poultry farming systems. The immune status of chickens plays a pivotal role in shaping gut microbiota communities, as reflected in the relationship between leukocyte levels and microbial diversity. Chickens with elevated leukocyte counts generally exhibited reduced microbiota diversity compared with those with lower leukocyte levels. This relationship suggests that inflammatory immune responses suppress microbial diversity by selecting taxa that are immune-mediated stress [35]. However, it remains uncertain whether immune activation, such as increased leukocyte counts, disrupts the microbiome or whether dysbiosis itself triggers heightened immune responses. It is plausible that a bidirectional relationship exists, in which microbial imbalance and immune activation mutually reinforce each other. Interestingly, chickens with balanced hematological profiles, particularly in erythrocyte and leukocyte parameters, tended to harbor richer and more stable microbial communities in the small intestine. This observation suggests that hematological homeostasis makes a significant contribution to microbial diversity in this region. The active mucosal surface of the small intestine appears to benefit from this balance, allowing the proliferation of beneficial commensals while limiting dysbiosis.

### Hematological stability and microbiota complexity

Conversely, chickens with leukocyte levels within normal limits and stable erythrocyte-to-leukocyte ratios demonstrated more complex and stable microbiota, reflecting optimal immune function and balanced host-microbe interactions. Maintaining microbial balance in the digestive tract helps modulate immune responses mediated by immune cells [36]. In addition, maintaining erythrocyte stability and leukocyte differentials within the normal range is associated with increased resistance to pathogens. The results of this study indicate that the hematological values of IPB-D3 chickens remain within the normal range. This finding is consistent with Lestari *et al*. [37], who reported advancements in the immune system characteristics of IPB-D3 chickens compared with their parental line, IPB-D1, including higher IgY concentrations and improved leukocyte differentiation. Furthermore, rearing IPB-D3 chickens under different management systems did not result in any hematological disturbances, suggesting that the birds have stable physiological adaptation across varying environments. Chickens with stable hematological indices exhibited increased abundance of commensal phyla, such as Proteobacteria and Bacteroidetes, which may enhance their resistance to infection [38]. The dynamic relationship between the immune system and gut microbiota is shaped by multiple factors, including environmental conditions and rearing systems, where more natural and diverse environments foster a balanced intestinal microbial ecosystem [39]. Thus, maintaining physiological erythrocyte and leukocyte values is a valuable indicator of immune competence and productive efficiency in poultry health management.

### Species-level shifts in gut microbial communities

The composition of chicken gut microbiota is shaped by both rearing systems and immune status, as reflected in the variation in dominant bacterial species across groups. In the IN system, *G. anatis* dominated (31%) in chickens with HL counts, a species associated with respiratory and reproductive pathologies in poultry [40]. These findings align with a previous study by Allahghadry *et al*. [41] that also identified *G. anatis* in both broiler and layer chickens from Iran. FR chickens with LL levels showed a predominance of *B. caecigallinarum* (16%), which may contribute to enhanced fiber fermentation and SCFA production that supports intestinal health. In contrast, FR chickens with HL levels were dominated by *L. aviarius* (31%), which exerts probiotic effects by promoting gut health through lactic acid production and microbiota balance [42]. In contrast, FR chickens with LL counts were predominantly colonized by *B. caecigallinarum* (16%), a species potentially involved in fiber degradation and SCFA production that supports intestinal health. This bacterium has also been commonly identified in indigenous Indonesian chickens [43]. One possible mechanism underlying this pattern is that elevated leukocyte levels, indicative of an inflammatory immune response, may disrupt the integrity of the intestinal mucosal barrier, thereby facilitating colonization by opportunistic pathogens such as *G. anatis*. In contrast, the dominance of *B. caecigallinarum* under conditions of lower leukocyte counts likely reflects a more stable, non-inflammatory intestinal environment that supports the growth of commensal microbes capable of producing SCFAs, contributing to gut health and homeostasis. These results suggest that both immune status and rearing system exert significant influence on gut microbiota composition in IPB-D3 chickens. This underscores the importance of considering immune factors in poultry management strategies to support gut microbial balance and overall health. Higher microbial diversity in FR systems provides physiological benefits, including enhanced immune resilience and digestive efficiency, thereby improving production performance [44]. In contrast, the dominance of *G. anatis* in the IN system under HL conditions may indicate increased health risks, as opportunistic pathogens tend to thrive in low-diversity microbiota environments, increasing susceptibility to infections [40]. The presence of *L. aviarius* in FR chickens suggests probiotic potential through the production of bioactive metabolites that suppress pathogenic species and promote microbiota stability, and this species is commonly found in various types of chickens as well as in turkeys [39]. These findings confirm that rearing strategies have a significant influence on the gut microbial community, directly affecting poultry health. Therefore, adopting FR models with optimized environmental parameters may serve as an effective strategy to enhance poultry health and reduce the reliance on antibiotic interventions. Diversification of microbiota is essential for enhancing infection resistance and livestock productivity.

### Practical implications and future directions for sustainable poultry farming

Enhancing the diversity of the gut microbiota through FR rearing strategies offers a sustainable path forward in poultry farming by reducing the reliance on antibiotics and strengthening the natural immune defenses of birds. Greater microbial diversity is also associated with improved fiber fermentation and nutrient utilization efficiency, resulting in better production performance [45]. Furthermore, the FR system enhances meat quality by promoting a more balanced gut microbiota profile, which in turn influences nutritional value and food safety through microbe–metabolite interactions within the chicken’s body [46]. However, further research is needed to fully understand the complex interactions between gut microbiota, immune status, and production outcomes under varying environmental and management conditions, in order to optimize sustainable poultry production practices. Potential limitations, such as pooling of intestinal samples, which may have masked individual variation, environmental differences, including soil microbiota across rearing systems, and stress during sampling procedures, such as blood collection or intestinal dissection, may have influenced the microbial and immunological profiles observed and should be considered when interpreting the findings.

## CONCLUSION

This study demonstrates that rearing systems have a significant influence on the gut microbiota diversity, immune status, and physiological stability of IPB-D3 chickens. FR chickens exhibited significantly greater alpha diversity indices (Observed OTU, Chao1, Shannon, Simpson, Fisher) compared to intensively reared chickens, indicating a richer and more balanced microbial ecosystem. Beta diversity analysis confirmed that both the rearing system and leukocyte levels substantially affected microbial community structure. Notably, chickens with lower leukocyte counts showed more diverse and stable gut microbiota, while those with elevated leukocyte counts, particularly under IN systems, were dominated by opportunistic pathogens such as *G. anatis*. In contrast, *L. aviarius* and *B. caecigallinarum* were predominant in FR chickens, suggesting advantages related to probiotic activity and fiber fermentation. Hematological parameters remained within normal physiological ranges across all groups, confirming the adaptability and immunological robustness of the IPB-D3 line.

The findings underscore the practical significance of the rearing environment in influencing gut microbial composition and immune responses in poultry. FR systems support greater microbial diversity and reduce pathogen overgrowth, offering a practical pathway to enhance poultry health, feed efficiency, and reduce antibiotic dependency – key goals in sustainable animal production systems.

This study’s strengths include the use of full-length 16S rRNA sequencing through Oxford Nanopore Technology, which allowed accurate species-level taxonomic resolution, and the integration of immune profiling, providing insights into host–microbe interactions under different rearing systems. However, several limitations must be acknowledged. Pooling of intestinal samples may have masked individual variation. The absence of environmental microbiota data from the rearing environments, particularly soil or vegetation in the FR system, limits ecological interpretation. Additionally, sampling-related stress (e.g., from blood collection and dissection) may have impacted certain immunological and microbial parameters.

Future research should include metatranscriptomic and metabolomic analyses to assess microbial function and its relationship with host physiology. Longitudinal studies could provide insight into temporal shifts in microbiota and immune function across different production phases. Tailored dietary interventions or probiotics based on rearing conditions may further optimize gut health and productivity in IPB-D3 and similar local breeds.

In conclusion, this study reinforces the pivotal role of the gut microbiome in poultry health and emphasizes how rearing systems modulate host–microbe–immune interactions. The IPB-D3 chicken, with its physiological resilience and adaptable microbiota, represents a strong candidate for sustainable poultry production. FR systems promote greater microbial diversity and immune stability, resulting in improved performance and reduced disease risk. Integrating microbiome-informed strategies into poultry management could significantly advance animal welfare, productivity, and environmental sustainability.

## DATA AVAILABILITY

All the generated data are included in the manuscript.

## AUTHORS’ CONTRIBUTIONS

CB, IK, and CS: Conceptualized and designed the study and reviewed and edited the manuscript. CS: Guided and supervised the study. WK, CB, and IK: Methodology development, data analysis, and interpretation of the results. WK: Drafted the original version of the manuscript. CB and CS: Performed validation and investigation. WK and IK: Managed the administrative aspects of the project. All authors have read and approved the final version of the manuscript.
